# Comments about the *IJMS* Special Issue: Molecular Interactions and Mechanisms of COVID-19 Inhibition

**DOI:** 10.3390/ijms23179607

**Published:** 2022-08-25

**Authors:** Francesco Caruso, Miriam Rossi

**Affiliations:** Department of Chemistry, Vassar College, Poughkeepsie, NY 12604, USA

The unprecedented COVID-19 pandemic showed up during the latter part of 2019 in Wuhan, China. From December 2019 to the summer of 2020, both of us were in Italy, which was the first European country overwhelmed by the COVID-19 infection, although other European countries, the USA, and other countries later followed a similar pattern. The Italian government took drastic measures to contain the virus but unexpected health impacts devastated the country (close to 200 health professionals died in the first 2 months of this health emergency) [1]. 

Scientifically, all aspects the virus and how to mitigate its health consequences were of immediate concern, and in April 2020, a preprint appeared (later published) describing a Dutch study that demonstrated that low levels of vitamin K (a quinone) worsened the condition of COVID-19 patients [2]. Since earlier research in our laboratory described antioxidant activities of quinone natural products, we thought that compounds containing a quinone moiety could be sensitive to COVID-19 and subsequently published two related papers [3,4]. 

Indeed, celastrol, a methide quinone natural compound, was reported as an inhibitor of (SARS-CoV) 3CL Protease in 2010 [5], and this study motivated us in examining the active site of this important target. We focused our attention on Cysteine-145 at the active site and described a potential inhibitory mechanism of Main-Pro [6]. We were convinced that research into the infectious mechanism, the spread of transmission, and potential treatments and cures required interdisciplinary efforts. Therefore, we decided to contact *IJMS* to promote this COVID-19 Special Issue.

This Special Issue includes a large variety of COVID-19-related subjects for which their aims are to propose mechanisms of action of the SARS-CoV-2 virus: Three reports describe the inhibition of proteins involved in virus reproduction such as the main protease, papain-like protease, and TMPRSS2. Three studies deal with a decrease in the extensive cytokine storm presented during the COVID-19 infection by (1) commonly used anti-inflammatory drugs’ inhibitory action on Caspase-1 (a trigger of pro-inflammatory cytokines); (2) phytoestrogens on estrogen receptors; and (3) acetylsalicylic acid derivatives. There are four investigations dealing with ACE-2 proteins and two reviews. A study shows the inhibition of replications on multi SARS-CoV-2 variants by sulfated polysaccharides extracted from red seaweed, whereas another related study shows inhibitions by lectin. The published reprints are available at: http://www.mdpi.com/journal/ijms/special_issues/covid-19_Inhibition. Accessed on June 16 2022.
